# Supplementary material for: Probing the evolutionary robustness of two repurposed drugs targeting iron uptake in Pseudomonas aeruginosa
Source: Evol Med Public Health. 2018 Sep 10;2018(1):246–59. doi: 10.1093/emph/eoy026 (PMC6234326; doi:10.1093/emph/eoy026)
Supplement: Supplementary Data [file eoy026_supp.docx]

**Supporting information captions**

**Supplementary Figure S1. Human Serum is an iron limited media.** To show that pyoverdine production is beneficial in human serum, we compared growth of *P. aeruginosa* PAO1 wildtype and the siderophore-deficient mutant PAO1 *ΔpvdD*, in pure human serum or human serum supplemented with the strong iron chelator human apo-transferrin (100 μg/ml) and its co-factor NaHCO_3_ (20 mM). When adding only 50 mM HEPES to buffer the medium at physiological pH (first panel) we observed that growth of the siderophore mutant was significantly reduced compared to the wildtype (Welch’s t-test, *t*_9_= 7.31, *p* < 0.0001), confirming that human serum is an iron-limited media. When we increased iron limitation (second panel), we found that overall growth decreased compared to pure human serum (ANOVA, growth in human serum + transferrin: *t*_21_= -22.35, *p* < 0.0001), but that the wildtype PAO1 still grew significantly better compared to the siderophore mutant. (Welch’s t-test, *t*_10_= 3.30, *p* < 0.0086), confirming that the siderophore pyoverdine is important for iron scavenging in human serum. All growth data are scaled relative to the wildtype growth in human serum supplemented with HEPES 50 mM. Error bars denote standard errors of the mean across 6 replicates.

**Supplementary Figure S2. Gallium and flucytosine affect growth and pyoverdine production of *P. aeruginosa* in plain human serum, not supplemented with additional transferrin**. To test the possibility that the two drugs affect growth and pyoverdine differently in human serum with and without additional transferrin, we performed dose-response curves in plain human serum, without adding the iron-chelator. We found that both anti-virulence drugs reduce growth of bacterial cultures (A, B) and pyoverdine production (C, D) in a dose-dependent manner, similarly to the reduction observed in human serum with transferrin (Fig. 1). As PAO1 has higher growth potential in plain serum (Supplementary Fig. S1A), the inhibitory effect of the two drugs at high concentrations was more pronounced than in serum with transferrin. Error bars denote standard errors of the mean across 6 replicates. Dose-response curves were fitted using a spline fit.

**Supplementary Figure S3. Growth and pyoverdine production profiles of evolved single clones under treatment regimes**. We were interested in examining the variation in growth and pyoverdine production profiles among evolved clones. For that purpose, we streaked out all evolved cultures on LB agar, and picked 5 random clones per evolved population. Overall, we isolated 160 clones, 40 clones per treatment. Single clones were tested under the treatment regime experienced during experimental evolution, and growth and pyoverdine production were measured after 24 hours. Panels show growth and pyoverdine production of evolved clones relative to the untreated wildtype (grey line) under gallium (A, C) and flucytosine treatment (B, D). We found heterogeneous growth and pyoverdine patterns under all treatment regimes, suggesting diversification in evolving populations. Data points show means across three independent replicates. Dashed lines depict the mean growth or the mean pyoverdine production of the ancestral strain under treatment. Labeled single clones marked in red refer to the clones used for in-depth analysis and sequencing.

**Supplementary Figure S4**. **Pyoverdine non-producer strains evolved in human serum with transferrin in the no-drug control treatment**. The strong iron limitation in human serum with transferrin could alone exert selective pressure during the experimental evolution, independently from the drug. This could affect the ability of the evolved clones to produce siderophore in response to the iron-limitation. To investigate these effects and control for general adaptation to low iron conditions, we isolated 40 clones from each treatment (gallium low, gallium high, flucytosine low; flucytosine high; no-drug control) and screened their ability to produce pyoverdine in human serum with transferrin. We observed that in the populations evolved without drug, pyoverdine-negative clones evolved at a frequency of approx. 25%. On the contrary, all clones from the drug treatments still produced pyoverdine. Each data point represents a single measurement per evolved clone. The black line denotes the average wildtype production level in the same assay and the grey area refers to the wildtype mean ± standard error of 20 replicates.

**Supplementary Figure S5: Evolved clones from the no-drug control treatment remain sensitive to both gallium and flucytosine**. We were interested in determining whether media adaptation could per se lead to reduced susceptibility to gallium and flucytosine. To test this, we picked 4 random clones from the no-drug evolved population and subjected them to a range of drug concentration, for both gallium and flucytosine, to measure if they still respond to the drug. Growth dose-response curves under treatment showed that all evolved clones were equally or more sensitive to both gallium (A-B) and flucytosine (E-F), compared to the ancestor PAO1. Similarly, pyoverdine dose-response curves showed that all clones were still affected by gallium (C-D). Under flucytosine, three clones, were slightly less sensitive to flucytosine compared to the ancestor (G-H), although they can still considered sensitive to the antimicrobial if compared to the resistant clones isolated from the flucytosine high or low treatment (Figure 3 G-H). Growth and pyoverdine production were measured after 24 h. For each clone, values are scaled relative to its performance in human serum without drugs. We used spline functions to fit dose-response curves, and used the integral (area under the curve) to quantify the overall dose response of each clone across the concentration gradient. Error bars denote standard errors of the mean across 6 replicates. Asterisks represent significance levels: * = p < 0.05; *** = p < 0.0001, based on linear model with df = 30.

**Supplementary Figure S6. Growth and pyoverdine production of evolved single clones in human serum with no treatment.** We quantified the growth (A) and pyoverdine production (B) of the 16 evolved single clones we used for in-depth analysis and whole-genome sequencing. Specifically, we grew the clones for 24 hours in human serum and compared their performance to the ancestor wildtype in absence of the treatments. This allowed us to test for media adaptation. (A) We observed that some of the clones from both treatments showed significantly improved growth compared to the ancestral wildtype. This was the case for clones GL1 (*t*_43_ = 2.04, *p* = 0.0475), GL_4 (*t*_43_ = 2.04, *p* = 0.0475), GH_1 (*t*_43_ = 3.13, *p* = 0.0125), FL_1 (*t*_43_ = 5.88, *p* < 0.0001) and all clones evolved under high flucytosine (FH_1: *t*_43_ = 3.09, *p* = 0.0054; FH_2:, *t*_43_ = 3.03, *p* = 0.0054; FH_3: *t*_43_ = 4.38, *p* = 0.0002; FH_4, *t*_43_ = 2.82, *p* = 0.0071). Interestingly, two of the clones evolved under gallium low treatment showed reduced growth compared to PAO1 (GL_2: *t*_43_ = -3.35, *p* = 0.0033; GL_3: *t*_43_ = -3.36, *p* = 0.0033). (B) Regarding per capita pyoverdine production (pyoverdine fluorescence divided by growth), we found that two clones showed increased levels of pyoverdine production under gallium treatment (GL_1: *t*_43_ = 2.79, *p* = 0.0200; GL_4: *t*_43_ = 2.69, *p* = 0.0200), and three clones of the flucytosine high treatment did so too (FH_1: t_43_ = 2.43, p = 0.0356; FH_2: *t*_43_ = 2.29, *p* = 0.0356; for FH_4: *t*_43_ = 2.35, *p* = 0.0356). In contrast, there were also a number clones with significantly reduced pyoverdine production compared to PAO1 (GH_1: *t*_43_= -3.14, *p* = 0.0045; GH_3: *t*_43_= -3.10, *p* = 0.0045; GH_4: *t*_43_= -3.46, *p* = 0.0045; FL_4: *t*_43_= -2.02, *p*=0.0488). All data are scaled relative to the ancestor wildtype. Error bars denote standard error of the mean across 6 replicates. Asterisks represent significance codes (* = p<0.05; ** = p<0.001, *** = p<0.0001 based on ANOVA, corrected for multiple pairwise comparison with the false discovery rate method).

**Supplementary Figure S7. Alteration of the fluorescent properties of pyoverdine when bound to gallium**. The fluorescent signal of pyoverdine becomes inflated when gallium binds to it [19]. To take this signal bias into account, we quantified the bias in fluorescence signal as a function of gallium concentration. Specifically, we supplemented iron-limited human serum with 200 μM of purified pyoverdine across a range of gallium concentrations (8 to 512 μM, as used for the main experiments). We found that the signal bias can be explained by a 3 parameters-logistic function. We used this function to correct for signal bias in all our analyses.

**Supplementary Table S1. List of control strains used in this study.**

**Supplementary Table S2. Differences to the PAO1 reference genome shared among all sequenced evolved clones and the ancestor wildtype.**

**Supplementary Table S3. Effects of mutation on the Upp protein sequence of evolved single clones**
