# Supplementary figures and images for: Probing the evolutionary robustness of two repurposed drugs targeting iron uptake in Pseudomonas aeruginosa
Source: Evol Med Public Health. 2018 Sep 10;2018(1):246–59. doi: 10.1093/emph/eoy026 (PMC6234326; doi:10.1093/emph/eoy026)

Additives to human serum

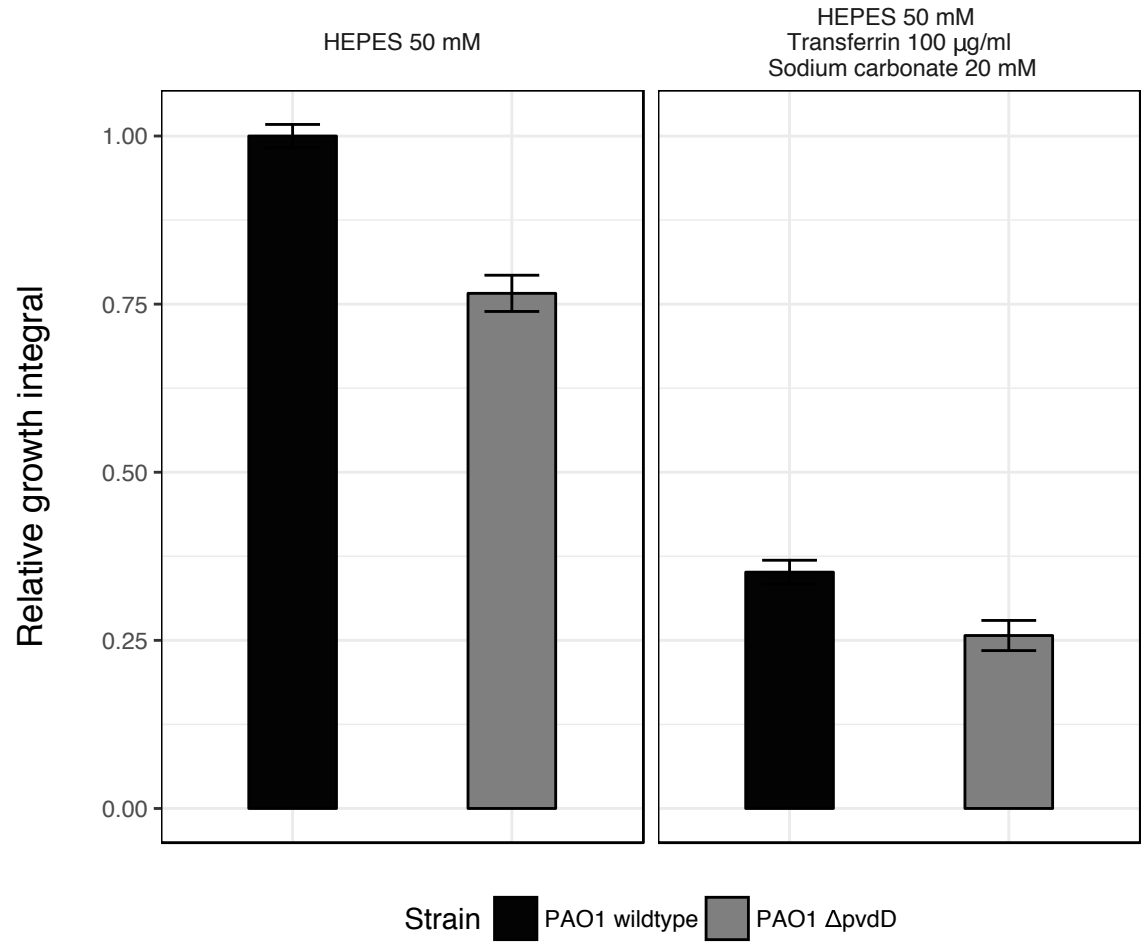

Supplementary Figure S1

Supplement: Supplementary Figure S1 [file eoy026_supp_fig_s1.pdf]

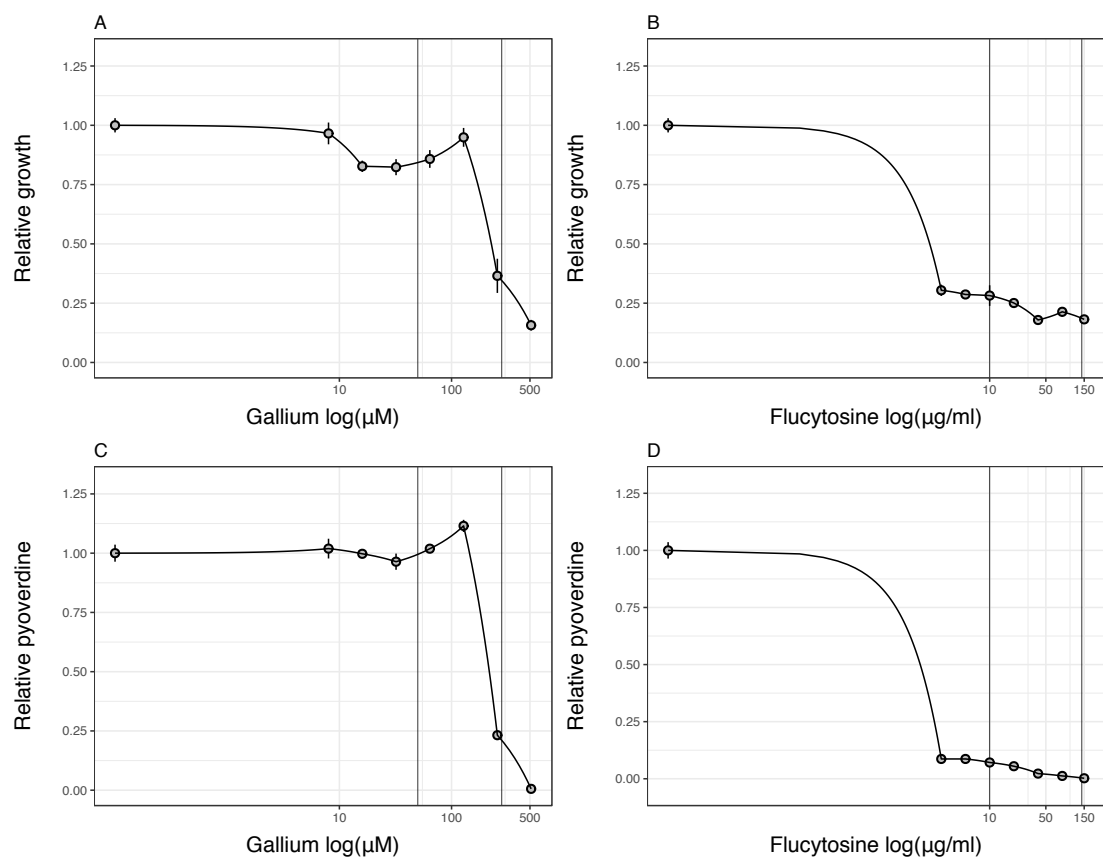

**Supplementary Figure S2**

Supplement: Supplementary Figure S2 [file eoy026_supp_fig_s2.pdf]

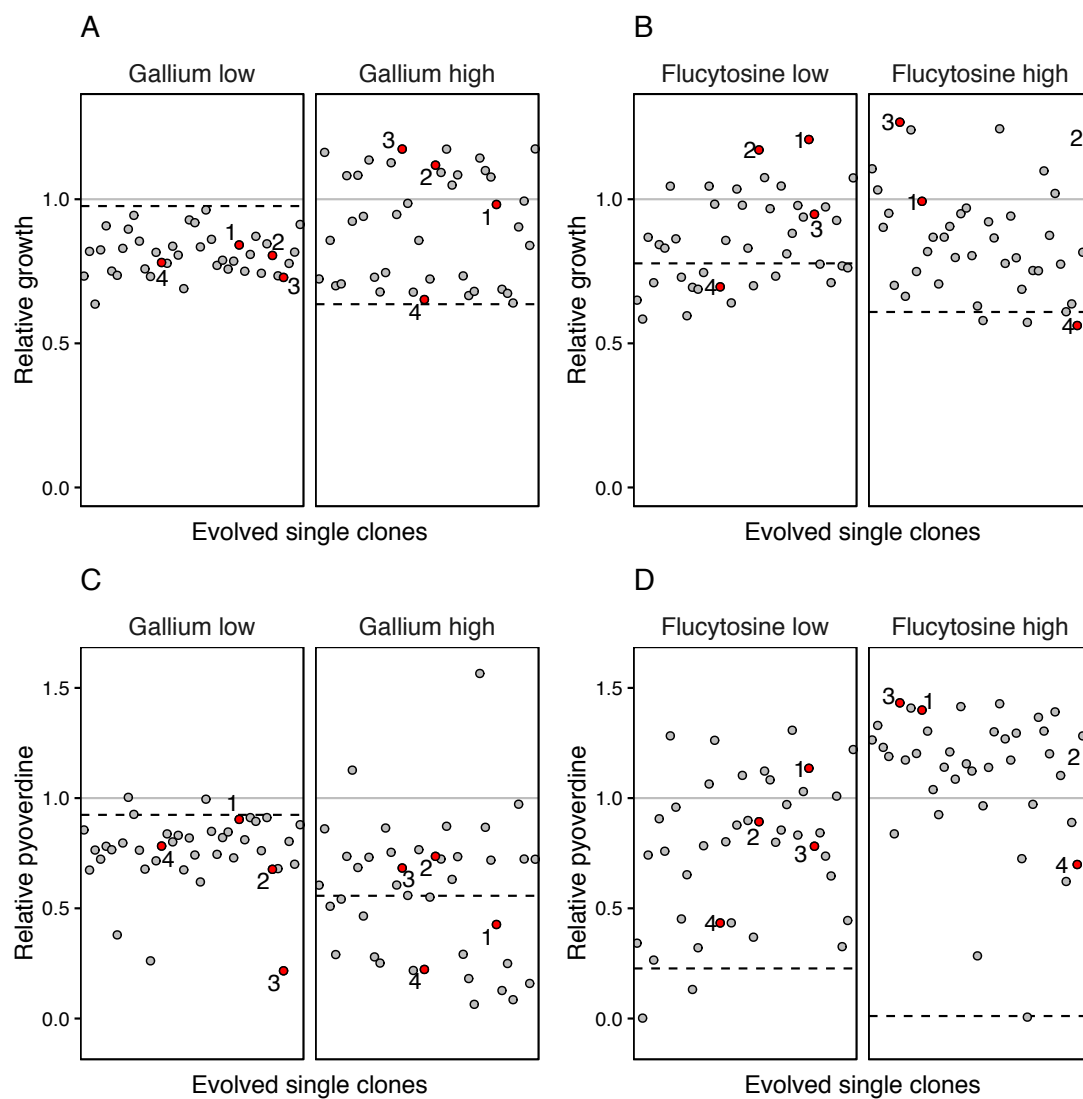

**Supplementary Figure S3**

Supplement: Supplementary Figure S3 [file eoy026_supp_fig_s3.pdf]

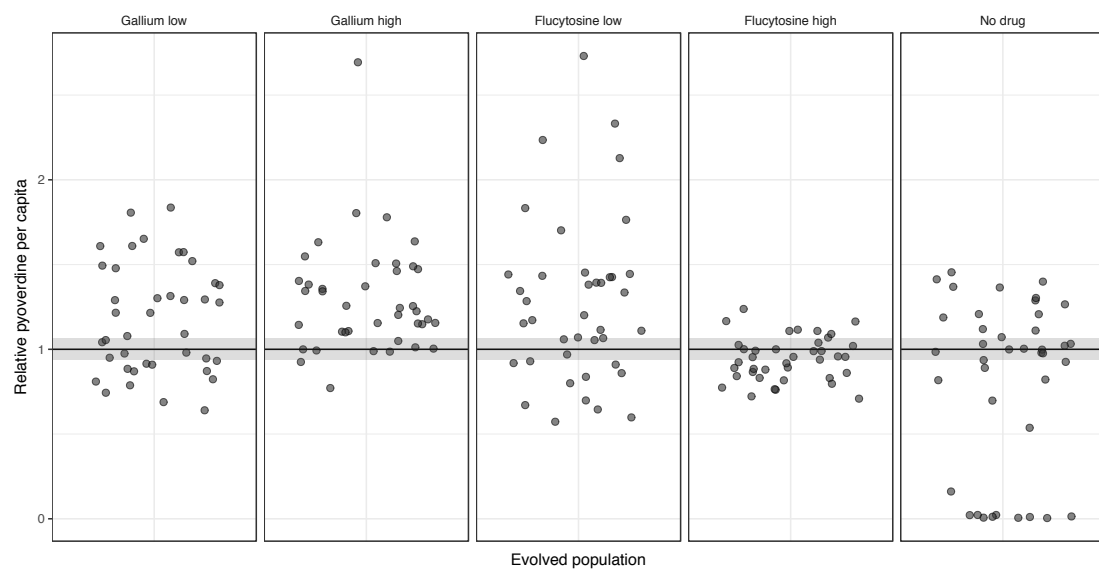

**Supplementary Figure S4**

Supplement: Supplementary Figure S4 [file eoy026_supp_fig_s4.pdf]

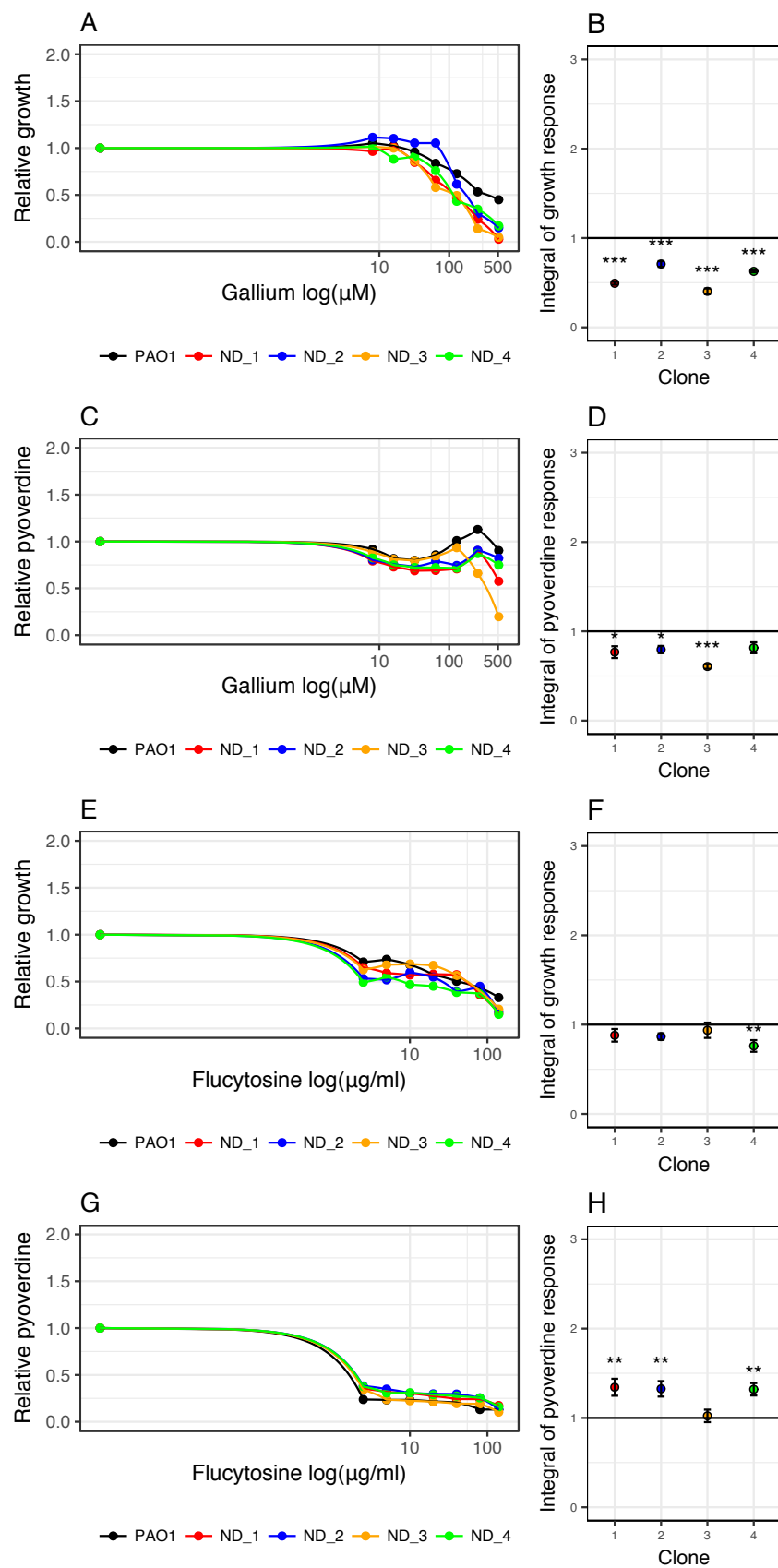

Supplementary Figure S5

Supplement: Supplementary Figure S5 [file eoy026_supp_fig_s5.pdf]

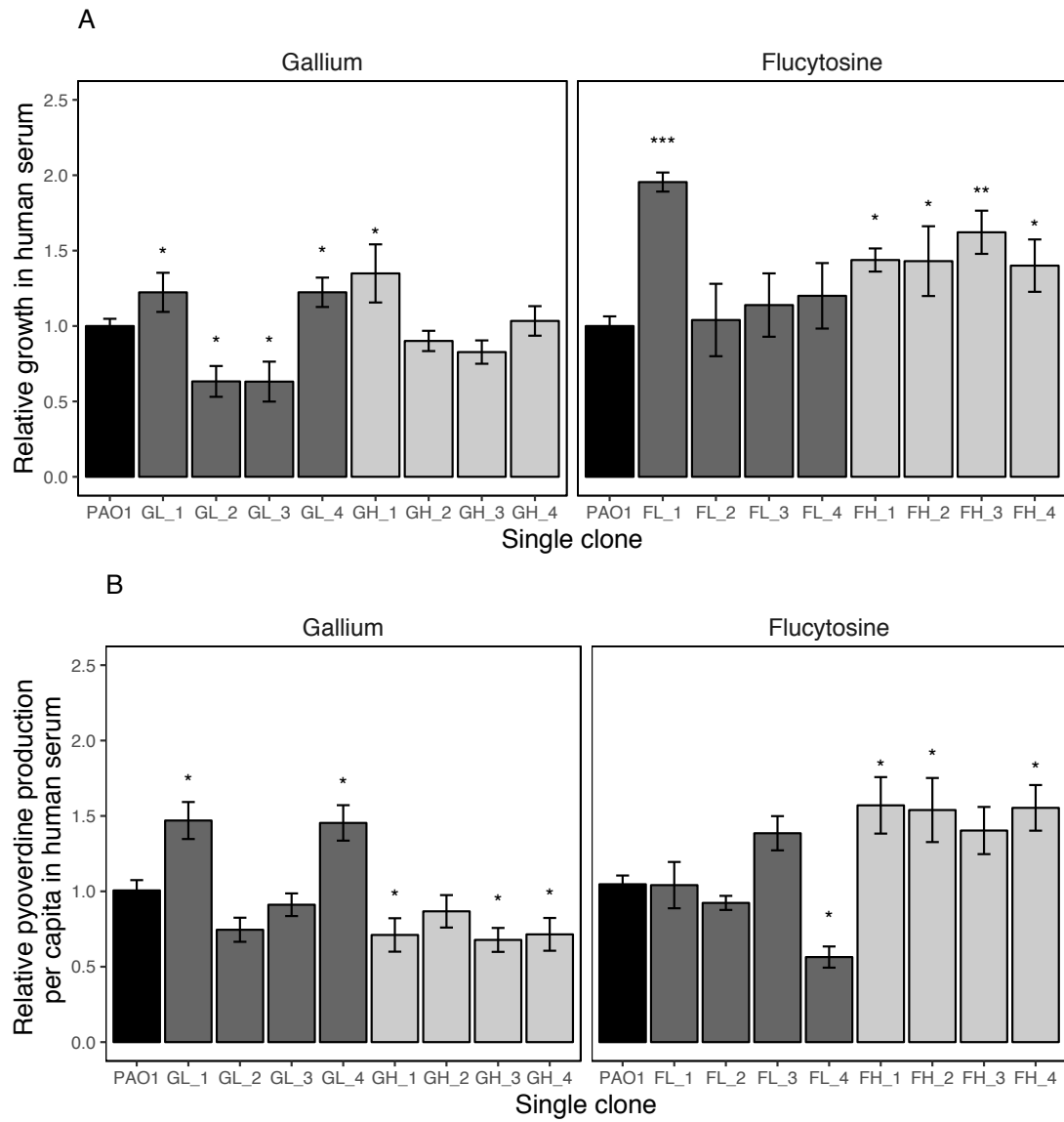

**Supplementary Figure S6**

Supplement: Supplementary Figure S6 [file eoy026_supp_fig_s6.pdf]

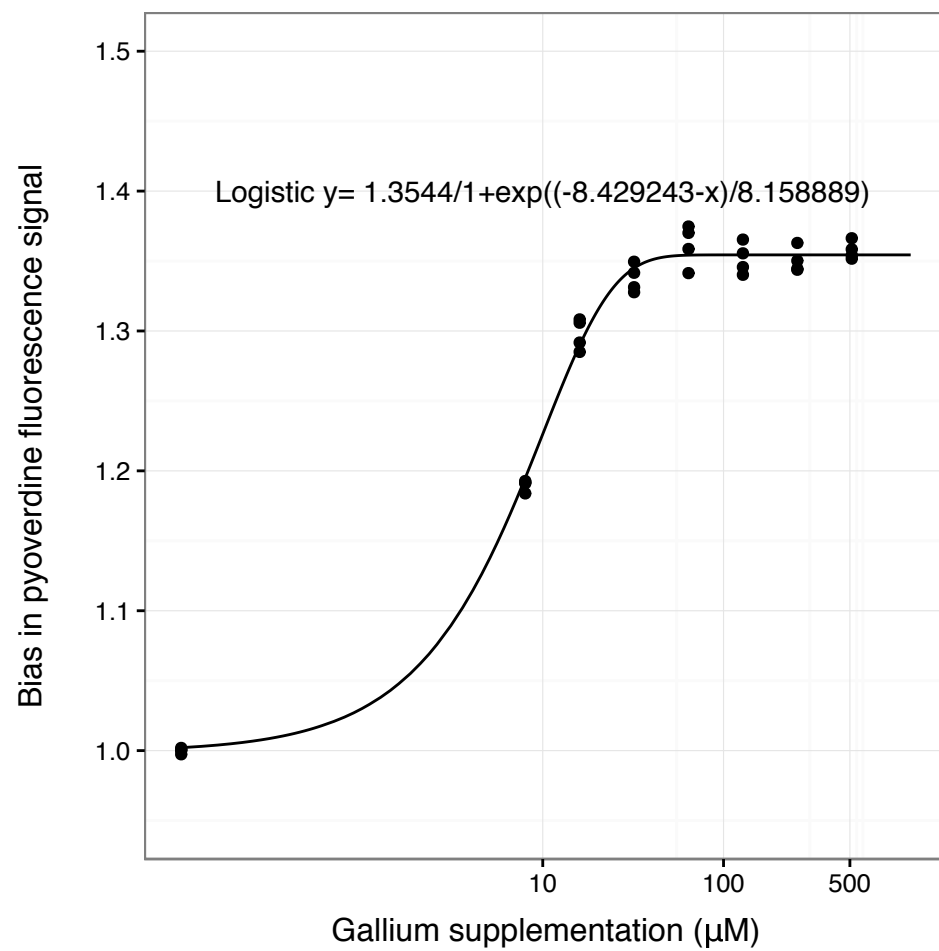

**Supplementary Figure S7**

Supplement: Supplementary Figure S7 [file eoy026_supp_fig_s7.pdf]
