# Supplementary material for: Probing the evolutionary robustness of two repurposed drugs targeting iron uptake in Pseudomonas aeruginosa
Source: Evol Med Public Health. 2018 Sep 10;2018(1):246–59. doi: 10.1093/emph/eoy026 (PMC6234326; doi:10.1093/emph/eoy026)
Supplement: Supplementary Table S1 [file eoy026_supp_table_s1.pdf]

Supplementary Table S1

**Strain**	**Description**	**Source**
PAO1 ΔpvdD	Knock-out mutant, deficient for pyoverdine production	[1]
PAO1 ΔrhlR	Knock-out mutant, deficient for the production of the transcriptional regulator RhlR	S.Diggle strain collection, University of Nottingham
PAO1 ΔlasR	Knock-out mutant, deficient for the production of the transcriptional regulator LasR	S.Diggle strain collection, University of Nottingham
MPAO1	Wildtype strain	[2, 3]
MPAO1 Δupp (PW8832)	Transposon insertion mutant, deficient for Upp production	[2, 3]

Supplementary references:

1. Ghysels B, Dieu BTM, Beatson SA *et al.* FpvB, an alternative type I ferripyoverdine receptor of Pseudomonas aeruginosa. *Microbiology* 2004;**150**:1671–80.

2. Held K, Ramage E, Jacobs M *et al.* Sequence-Verified Two-Allele Transposon Mutant Library for Pseudomonas aeruginosa PAO1. *J Bacteriol* 2012;**194**:6387–9.

3. Jacobs MA, Alwood A, Thaipisuttikul I *et al.* Comprehensive transposon mutant library of Pseudomonas aeruginosa. *Proc Natl Acad Sci U S A* 2003;**100**:14339–44.
