# Supplementary material for: Probing the evolutionary robustness of two repurposed drugs targeting iron uptake in Pseudomonas aeruginosa
Source: Evol Med Public Health. 2018 Sep 10;2018(1):246–59. doi: 10.1093/emph/eoy026 (PMC6234326; doi:10.1093/emph/eoy026)
Supplement: Supplementary Table S2 [file eoy026_supp_table_s2.pdf]

Supplementary Table S2

Gene	Description	Position	Reference	Alternate PAO1	Alternate clones	Type
PA0159	Transcriptional regulator	183697	T	G	G	SNP
PA0148-PA0149	Intergenic region	169283	CG	CG	C	INDEL
PA0173	probable methyltransferase	197724	C	C	CG	INDEL
PA0366-PA0367	intergenic region	411125	AC	AC	A	INDEL
PA0369-PA0370	intergenic region	413850	T	C	C	SNP
PA0604-PA0605	intergenic region	413850	T	T	C	SNP
PA0668-rRNA104078	intergenic region	721611	C	C	T	SNP
PA0683	HxcY, type II protein secretion system complex	740419	G	GC	GC	INDEL
PA0748	hypothetical protein	816529	GCCC	GCCCC	GCCCC	INDEL
PA0812-PA0813	intergenic region	891099	AC	ACC	ACC	INDEL
PA1029	hypothetical protein	1116213	G	GC	GC	INDEL
PA1122	putative peptide deformylase	1215657	AGG	AGGG	AGGG	INDEL
PA1174	NapA, periplasmic nitrate reductase protein	1275766	GAA	GA	GA	INDEL
PA1327	probable protease	1440622	CAAA	CAA	CAA	INDEL
PA1332-PA1333	intergenic region	1445357	A	AG	AG	INDEL
PA1352-PA1353	intergenic region	1467482	A	AGC	AGC	INDEL
PA1459	probable methyltransferase	1589438	G	C	C	SNP
PA1685	MasA,enolase-phosphatase E-1	1835045	G	GC	GC	INDEL
PA1982-PA1983	intergenic region	2169348	AGG	AGGG	AGGG	INDEL
PA2000-PA2001	intergenic region	2186927	GGCG	GCGCG	GCGCG	INDEL
PA2006-PA2007	intergenic region	2195457	GCCCCC	GCCCCCC	GCCCCCC	INDEL
PA2046-PA2047	intergenic region	2239555	A	AG	AG	INDEL
PA2046-PA2047	intergenic region	2239547	T	G	G	SNP
PA2127-PA2128	intergenic region	2342110	C	C	CT	INDEL
PA2139	hypothetical protein	2355771	A	A	AG	INDEL
PA2141	hypothetical protein	2356681	GCCC	GCC	GCC	INDEL
PA2300-PA2301	intergenic region	2532046	G	GC	GC	INDEL
PA2400	PvdJ, pyoverdine biosynthetic process	2669175	G	C	C	INDEL
PA2452	hypothetical protein	2753522	G	GC	GC	INDEL

PA2492	MexT, transcriptional regulator	2807693	TCGGCCAGCCGGCCAGCCGGCCA	TCGGCCAGCCGGCCA	TCGGCCAGCCGGCCA	INDEL
PA2492	MexT, transcriptional regulator	2807982	T	A	A	SNP
PA2492	MexT, transcriptional regulator	2808180	C	A	A	SNP
PA2668	hypothetical protein	3016844	GC	GCC	GCC	INDEL
PA2727	hypothetical protein	3083196	A	AG	AG	INDEL
PA3503-PA3504	intergenic region	3919508	G	G	GC	INDEL
PA3969-PA3970	intergenic region	4448855	C	C	G	SNP
PA3969-PA3970	intergenic region	4448856	G	G	C	SNP
PA3760	N-Acetyl-D-Glucosamine phosphotransferase system transporter	4212201	A	G	G	SNP
PA3877	nitrite extrusion protein 1	4344266	A	G	G	SNP
PA4059	hypothetical protein	4539468	GC	GCC	GCC	INDEL
PA4280.2-PA4280.3	intergenic region	4789053	C	CA	C	INDEL
PA4341	probable transcriptional regulator	4869855	T	G	G	SNP
PA4360	hypothetical protein	4888194	A	AG	AG	INDEL
PA4394	conserved hypothetical protein	4924552	C	G	G	SNP
PA4394	conserved hypothetical protein	4924553	G	C	C	SNP
PA4496	probable binding protein component of ABC transporter	5033101	G	GC	GC	INDEL
PA4499-PA4500	intergenic region	5036891	A	C	C	SNP
PA4526-PA4527	intergenic region	5071543	AACTG	AACTG	A	INDEL
PA4550	FimU type 4 fimbrial biogenesis protein	5098663	AGGCC	AGGCC	A	INDEL
PA4874-PA4875	intergenic region	5472415	C	C	CG	INDEL
PA5024	conserved hypothetical protein	5655220	CCGGCGGCGGC	CCGGCGGCGGCGGC	CCGGCGGCGGCGGC	INDEL
PA5100	hutU, urocanase	5743461	C	G	G	SNP
PA5100	hutU, urocanase	5743462	G	C	C	SNP
PA5399	DgcB, Dimethylglycine catabolism	6079222	A	G	G	SNP
PA5418	sarcosine oxidase alpha subunit	6098781	G	C	C	SNP
PA5434	tryptophan permease	6115455	T	G	G	SNP
